# Supplementary material for: The Involvement of LAG-3positive Plasma Cells in the Development of Multiple Myeloma
Source: Int J Mol Sci. 2023 Dec 31;25(1):549. doi: 10.3390/ijms25010549 (PMC10778841; doi:10.3390/ijms25010549)
Supplement: Supplementary file 1 [file ijms-25-00549-s001.zip › ijms-2760031-supplementary.pptx]

## Slide 1
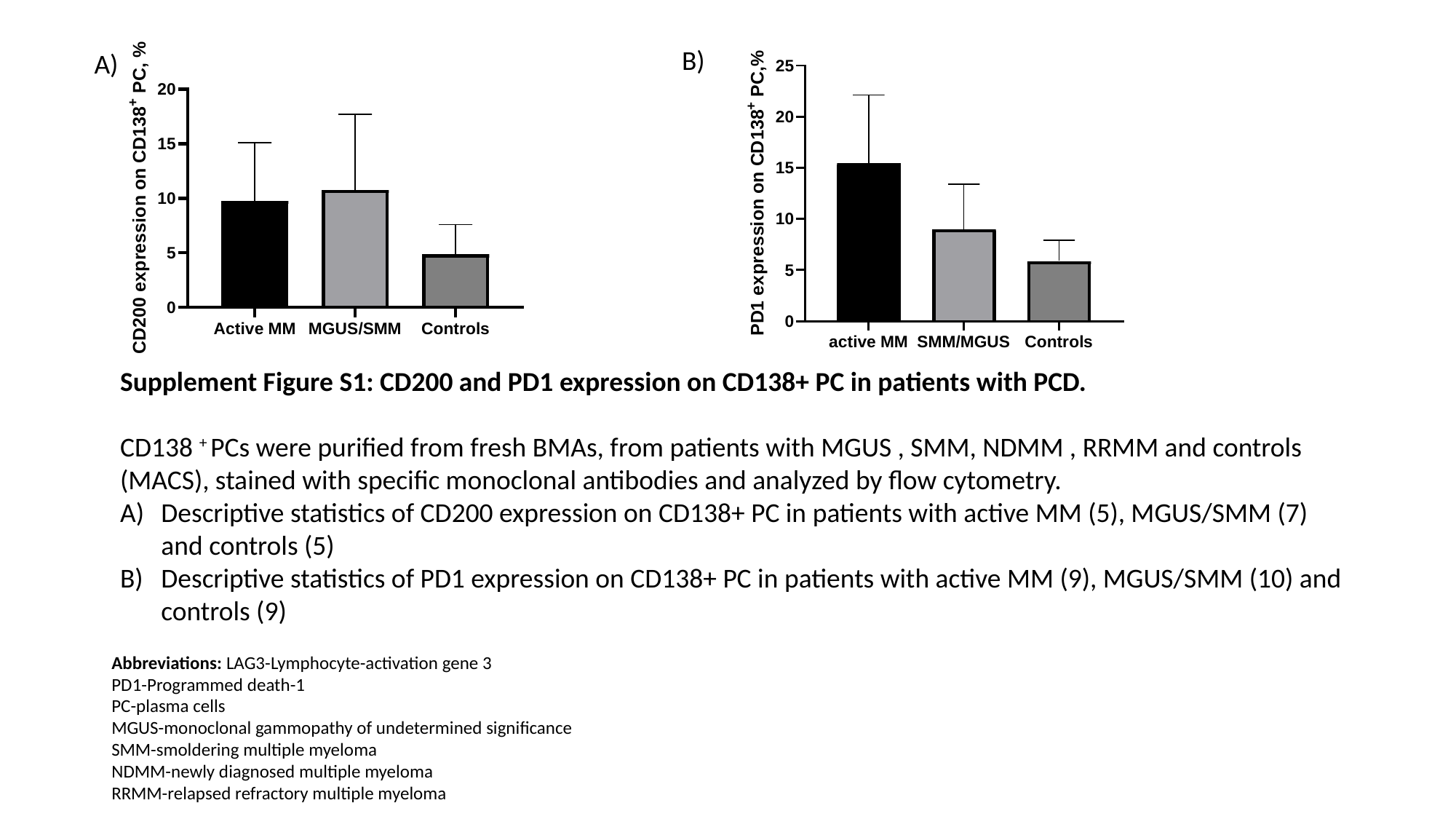

B)
A)
Supplement Figure S1: CD200 and PD1 expression on CD138+ PC in patients with PCD.
CD138 + PCs were purified from fresh BMAs, from patients with MGUS , SMM, NDMM , RRMM and controls (MACS), stained with specific monoclonal antibodies and analyzed by flow cytometry.
Descriptive statistics of CD200 expression on CD138+ PC in patients with active MM (5), MGUS/SMM (7) and controls (5)
Descriptive statistics of PD1 expression on CD138+ PC in patients with active MM (9), MGUS/SMM (10) and controls (9)
Abbreviations: LAG3-Lymphocyte-activation gene 3
PD1-Programmed death-1
PC-plasma cells
MGUS-monoclonal gammopathy of undetermined significance
SMM-smoldering multiple myeloma
NDMM-newly diagnosed multiple myeloma
RRMM-relapsed refractory multiple myeloma
